# Periosteal Osteoblastoma of the Pelvis: A Rare Case

**Published:** 2015-01

**Authors:** Swaroop Patel, Atul Agrawal, Rajesh Maheshwari, Vijendra D. Chauhan

**Affiliations:** Department of Orthopaedics, Himalayan Institute of Medical Sciences, Swami Ram Nagar, Dehradun, Uttarakhand, India

**Keywords:** Periosteal, Osteoblastoma, Pubic bone, Osteochondroma

## Abstract

Among the rare bone tumors, the osteoblastoma is a fascinating tumor. The rarity, the predisposition to occur in any bone and the diagnostic dilemma makes this infrequent tumor interesting. It is sporadically reported in the literature and what is rarer is its occurrence in the pelvis. The unusual location and inconclusive radiographic findings with diffused diagnostic evidences delays the management of benign osteoblastoma. We encountered a patient with benign osteoblastoma of the pubic ramus of right side. An excisional biopsy was performed. Peroperatively, the tumor appeared as oval, reddish brown, bony hard mass lying just over the cortex of the right pubic ramus and not breaching the cortex. Histopathological study revealed an osteoid rich lesion. Its presence in pubis must not be ignored and periosteal osteoblastoma should be considered as a differential diagnosis.

## Introduction

Among the rare bone tumors, the osteoblastoma is a fascinating tumor. The rarity, the predisposition to occur in any bone and the diagnostic dilemma makes this infrequent tumor interesting. Osteoblastoma forms less than 1% of the primary bone tumors.^1^ It is sporadically reported in the literature and what is rarer is its occurrence in the pelvis.

The unusual location and inconclusive radiographic findings with diffused diagnostic evidences delay the management of benign osteoblastoma. We encountered a patient with benign osteoblastoma of the pubic ramus of right side. 

## Case Report

**Figure 1:**
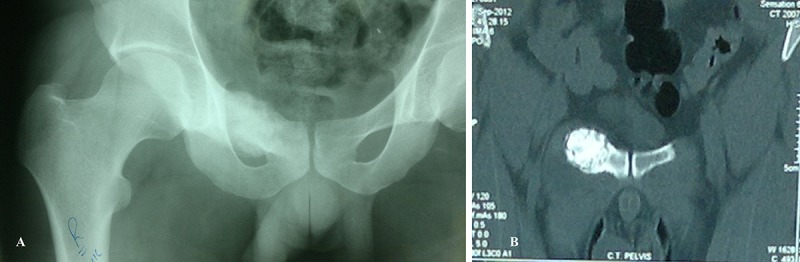
A) Radiograph showing cartilaginous growth in right superior ramus of Pubic Bone, suggestive of osteochondroma. B) Noncontrast computed tomography of pelvis showing exostosis of 3×2.5 cm involving the right superior pubic ramus.

After taking a written informed consent from the patient, the case was included in this research presentation. A 22-year-old male, complained of vague pain in the lower back with radiation to right inguinal region for the past one year. On clinical examination, a tender, bony hard swelling was palpated on the right side appearing to originate from the right pubic ramus. The movements of hip joint were normal and examination of the spine did not reveal any spinal pathology. Laboratory studies including blood counts, urine analysis, serum calcium, phosphorus, and alkaline phosphatase were within normal limits. Roentgenograms (figure 1A) showed cartilaginous growth in right superior ramus suggestive of osteochondroma. Non-contrast computed tomography of the pelvis also suggested of exestosis of 3×2.5 cm involving the right superior pubic ramus (figure 1B).

**Figure 2:**
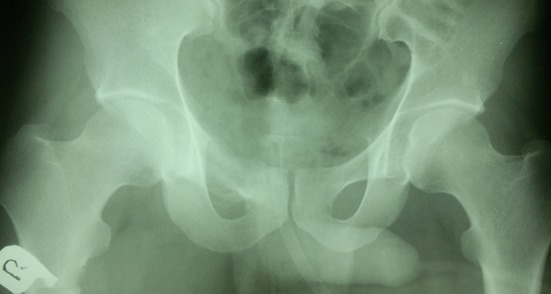
Postoperative radiograph of pelvis showing complete excision of the bony growth.

An excisional biopsy was performed. Peroperatively, the tumor appeared as oval, reddish brown, bony hard mass lying just over the cortex of the right pubic ramus and not breaching the cortex. No bone grafting was done, as the tumor resection did not hamper the strength of the bone (figure 2).

**Figure 3:**
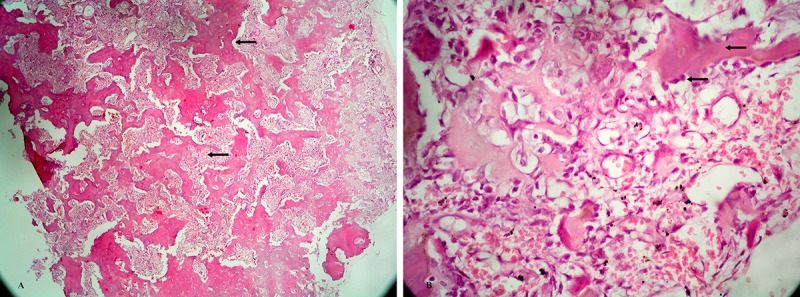
A) Microphotograph (×10, hematoxylin and eosin stain) showing numerous osteoid trabeculae of variable size, shape and separated by vascularised cellular stroma. 3) Microphotograph (×10, hematoxylin and eosin stain) showing some non-calcified osteoid. All are surrounded by plump osteoblast and the stroma is highly vascularised

Histopathological study revealed an osteoid rich lesion. The osteoid trabeculae were variable in size and thickness and were covered by plump osteoblast, which were also seen as a collection in between the osteoid trabeculae. Some osteoid was partially calcified. The inter-osteoid space contained many dilated capillaries, a few scattered osteoclast, and focal free collection of blood. Mitotic figures were absent and there was no necrosis. No cytological evidence of malignancy was noted. Normal periosteum and bone was seen on one side of the lesion without any evidence of infiltration (figure 3A, 3B).

Postoperative wound healing was satisfactory and the preoperative pain and discomfort disappeared. 

## Discussion

Osteoblastoma generally occurs in young individuals and more commonly in males.^2^ The tumor grows slowly with few symptoms, but aggressive lesions may cause soft tissue oedema, joint stiffness, and contracture.^3^ The vast majority of cases are intra-osseous (medullary) but a small percentage occurs on the surface of the bone as periosteal (peripheral) osteoblastoma. The axial skeleton, particularly the posterior elements are the most favorable sites of the osteoblastoma and account for 40% of the total, followed by those occurring in the long bones of the lower extremity, which account for 20%.^4^ Other uncommon sites of osteoblastoma are talus, scapula, cranium, distal femur and pelvis.^5^^-^^7^ Benign osteoblastic tumors of the pelvis are rarely encountered and in most instances they involve the acetabular area.^8^ The periosteal form of the tumor is highly unusual. No case of periosteal osteoblastoma of pelvis is reported in the English literature. We report a case of periosteal osteoblastoma in pubic bone as it is extremely rare. Peylan et al. reported a case of osteoblastoma occurring in the pelvis; the tumor involved the superior pubic ramus, the superior part of the ischium, and the inferior part of the acetabulum.^7^

The primary complaint in osteoblastoma is pain, characterized as dull aching or nagging type. Symptoms greatly depend upon the location and size, as in the spine it may produce deformity like scoliosis and may even produce neurological deficit. 

Osteochondroma could resemble as either osteoid osteoma or osteogenic sarcoma. In most of the cases reported earlier, the initial evaluation had suggested of malignancy but later proved to be benign. The rapid production of osteoid cells may give rise to a histological confusion and an erroneous diagnosis in the past has resulted in needless amputations.^5^

Osteoid Osteoma is differentiated from osteoblastoma and osteosarcoma by the size and quality of the growth. Osteoblastoma does not have either a nidus or the typical sclerosis, which surrounds the ordinary osteoid osteoma. The roentgenographic appearance of the osteoblastoma is inconsistent, although periosteal osteoblastoma may radiologically appear as osteochondroma. However, exostosis-like appearance is not common in periosteal osteoblastoma, but it should be differentiated from sessile type osteochondroma. Computed tomography is an invaluable tool; MRI and bone scan may add to the information.

In the present case, the tumor was reported as osteochondroma. The size of the tumor was 5×3 cm and appeared as hemorrhagic reddish brown with gritty feeling. Histologically, the tumor was made of newly formed osteoid associated with marked proliferation of plump osteoblast, with numerous vascular spaces in the stroma. Osteoid osteoma was ruled out as the location of the tumor was over the surface of the bone and was larger in size than the osteoid osteoma. Periosteal osteosarcoma was excluded as the tumor had no mitotic activity, necrosis, infiltration and had uniform nuclei. Due to the large number of plump osteoblasts, the tumor was considered aggressive osteoblastoma although much fibrous tissue was lacking.

The treatment of an established osteoblastoma is intralesional curettage and packing of the defect with bone graft. Lichtenstein described the removal of only the central ossified part without curettage^9^ but wide resection if feasible, is not contraindicated.

Rarely a benign osteoblastoma is reported to transform into malignancy.^10^^-^^12^ However, a close follow up of longer duration is advisable. The microscopic findings of the recurring lesion may show a definite increase in cellularity and the size of the nuclei of the osteoblasts, but these changes are not considered as malignant.^10^

## Conclusion

Periosteal osteoblastoma is a rare subtype of osteoblastoma. Its presence in pubis must not be ignored and periosteal osteoblastoma should be considered as a differential diagnosis. 
